# Developmental adverse effects of trace amounts of lead: Evaluation using zebrafish model

**DOI:** 10.3389/fphar.2022.1014912

**Published:** 2022-09-21

**Authors:** Yuta Komoike, Masato Matsuoka

**Affiliations:** Department of Hygiene and Public Health, Tokyo Women’s Medical University, Tokyo, Japan

**Keywords:** zebrafish, lead, development, oxidative stress, endoplasmic reticulum stress, DOHAD

## Abstract

Lead (Pb) is widely used as a raw material for various daily necessities in human civilization. However, Pb is a major toxicant and Pb poisoning has long been a global health concern. A large body of evidence has revealed that exposure to Pb causes a variety of adverse health effects. Meanwhile, experimental studies on the developmental effects caused by trace amounts of Pb remain to be fully conducted. Therefore, we aimed to provide direct experimental evidence of the adverse developmental effects of Pb exposure below the occupational regulatory standard concentrations using a zebrafish model. We also attempted to investigate the cellular stress response caused by such a trace amount of Pb at the individual level. Fertilized zebrafish eggs were exposed to 100 ppb Pb from 6 to 72 h post fertilization (hpf), the developmental period included within the mammalian implantation to birth. The embryos exposed to Pb did not show superficially evident morphological alterations or differences in viability compared with the controls until 72 hpf; however, they hatched earlier and were significantly shorter in body length than the controls at 48 and 72 hpf. Larvae that were exposed to Pb until 72 hpf and then cultured until 7 days post fertilization without Pb exhibited edema and inflation defects in the swim bladder. The reactive oxygen species level in the Pb-exposed embryos was similar at 24 hpf, slightly but significantly higher at 48 hpf, and lower than half that of the control at 72 hpf. Accordingly, the expression levels of oxidative stress response-related genes were analyzed, and five out of seven tested genes were upregulated in Pb-exposed embryos at 48 and 72 hpf. In addition, the endoplasmic reticulum (ER) stress related genes were upregulated at 48 hpf. These results indicate that exposure of embryos to trace amounts of Pb induces a transient increase in oxidative- and ER-stresses and results in weak hypotrophy and subsequent abnormalities later in development. Our findings may be key to understanding the total health effects of Pb exposure, and indicate that the zebrafish model is suitable for the investigation of developmental toxicity of pollutants such as Pb.

## Introduction

Lead (Pb) is an important heavy metal distributed ubiquitously across the Earth (Taylor et al., 2009). Owing to its properties such as ease of mining, refining, and processing, Pb has been widely used as a raw material for various products (industrial products, hobbies and amusement equipment, pigments, paints, etc.), all of which are closely tied to our daily lives. On the other hand, Pb has long been recognized as a toxicant and clinical cases caused by both occupational and environmental exposures to Pb have been well documented. Pb-induced health problems, which can be caused by both acute and chronic intoxication, are typified by three major clinical manifestations, that is, anemia, abdominal symptoms, and peripheral and central nervous system abnormalities (Skerfving and Bergdahl, 2007). In addition to these salient symptoms, reproductive toxicity, carcinogenicity, nephropathy, and effects on the cardio-vascular system have also been observed in patients with Pb poisoning (Skerfving and Bergdahl, 2007; ATSDR, 2020). Especially, many epidemiological studies have shown evident associations of blood Pb level (BLL) with fertility in both females and males and adverse effects on fetal development including growth retardation and low birth weight, even at very low levels (below 5 μg/dl) (NTP, 2012). Because of this immediate threat, regulations for Pb usage and regulatory standard concentrations are constituted in many countries. For example, the recommended limits for BLL are 200 μg/L by the American Conference of Governmental Industrial Hygienists (ACGIH), which was recently lowered from 300 μg/L (ACGIH, 2016), 300 μg/L for men and women over 45 years old by the German Commission for the Investigation of Health Hazards of Chemical Compounds in the Work Area (Deutsche Forschungsgemeinschaft, 2015), 25 μg/dl for women of reproductive age, 40 μg/dl for people 16–17 years old, and 50 μg/dl for all other employees by the United Kingdom Health and Safety Executive (HSE) (HSE, 2002), and 15 μg/dl by the Japanese Society for Occupational Health (JSOH) (JSOH, 2021). In response to increasing concern about the risks of Pb exposure, reduction in the usage of Pb is now promoted around the world (Europian Parlament and the Council, 2011; SAICM, 2015a; SAICM, 2015b; ECHA, 2021). Concomitantly, Pb-free technologies in industry are being developed; however, broad attention to adverse health effects induced by Pb is still highly required.

In addition to case reports and observational studies, experimental studies on Pb toxicity have been extensively conducted. Accordingly, the toxicokinetics of Pb, especially the molecular and cellular mechanisms underlying Pb toxicity, have been partly elucidated. In brief, Pb interacts with multiple cellular sites and can alter the functions of a wide variety of structural and functional proteins through binding to their sulfhydryl and carboxyl groups (Goering, 1993). Besides, Pb mimics calcium and other divalent cations and disturbs many cation-dependent cellular functions (Rabinowitz, 1991). Additionally, Pb increases cytotoxic reactive oxygen species (ROS) production via redox-inactive ways, leading to cellular damage (Jomova and Valko, 2011). Various symptoms of Pb poisoning have been experimentally reproduced using animal models. Chronic exposure to Pb via diet or drinking water that increases BLL to clinically relevant concentrations affects the hematopoietic system, peripheral and central nervous system, and cardiovascular system, as well as causing nephropathy, in rats and other experimental animals (ATSDR, 2020). Moreover, oral administration of Pb to rats and mice for various exposure periods at relatively high doses results in renal cancer and glioma (Boyland et al., 1962; Zawirska, 1981; Nogueira, 1987). In contrast, experimental studies on the adverse developmental effects, including growth retardation and/or low birth weight of neonates, caused by trace amounts of Pb remain to be fully conducted.

The zebrafish (*Danio rerio*) has been widely accepted as a common experimental animal model in various fields of biomedical research, and exhibits many advantageous features as a model animal in toxicology. For example, external fertilization and development enable easy control over exposure timing and duration, prolificacy eliminates the influence of genetic background on toxic effects, rapid development of embryos/larvae shortens the experimental period, and low-cost breeding of adult fish benefits researchers economically. Notably, zebrafish possess most of the biological systems that are highly conserved among all vertebrates and, consequently, can be used as an alternative to mammalian models despite their taxonomically distant relationships. In addition, zebrafish embryos and early larvae that do not reach the stage of starting independent feeding are exempt from the application of “the European Union directive on the protection of animals used for scientific purposes” (European Parliament and the Council, 2010). This point adds more value to zebrafish from the perspective of recent debate regarding the ethics governing animal experimentation.

In the present study, to provide direct experimental evidence for the adverse effects of Pb on embryonic/larval development below the occupational regulatory standard concentrations, we assessed the impact of Pb exposure on developing zebrafish at 100 ppb (10 μg/dl), which meets the current occupational regulatory requirements for BLL. We exposed fertilized zebrafish eggs to 100 ppb Pb during the developmental period included within mammalian implantation to birth, and its effects on viability, hatching, and growth were recorded. In addition, the Pb-exposed larvae were then raised without Pb and their morphology was observed later in development. Furthermore, we attempted to investigate the cellular stress response caused by such a trace amount of Pb at an individual level. For this purpose, alterations in ROS levels and the expression levels of oxidative stress response- and endoplasmic reticulum (ER) stress response-related genes following Pb exposure were investigated.

## Materials and methods

### Guidelines for animal experiments

Zebrafish were reared in a certified animal facility specifically for zebrafish approved by the institutional Animal Care and Use Committee (Tokyo Women’s Medical University). All experiments using zebrafish were designed according to the “Animals in Research: Reporting *in Vivo* Experiments (ARRIVE) guidelines” and the Institutional Ethical Code for Laboratory Animals and were approved by the committee (approved No.: AE21-094, AE22-108). The approval proofs are available upon request.

### Maintenance of adult zebrafish

Fish (RIKEN RW strain) were originally provided by the National Bio Resource Project Zebrafish (RIKEN Center for Brain Science, Wako, Japan) and were continuously bred in our zebrafish facility. Adult zebrafish were maintained under a 14/10-h light/dark cycle at 28.5°C in polystyrene cuboid tanks with a capacity of 4.5 L in a circulating water system. The total volume of circulating water in the system was approximately 120 L, and a portion of the water was constantly replaced by dechlorinated fresh tap water (5 L/h). The pH of the circulating water was naturally kept at 6.5–7.0 that was monitored once a week. Two females and three males from 6 months to 1 year of age (hereafter designated as the mating group) were reared per tank for breeding. Zebrafish were fed live *Artemia* larvae prepared at each feeding from high-quality *Artemia* cysts (Fujimoto Taiyodo, Kyoto, Japan) once every weekday and granule aqua-feed (Marubeni Nisshin Feed, Tokyo, Japan) three times per day. The dosage of *Artemia* larvae and granule feed was adjusted to be eaten completely within a few min of feeding. The health status of zebrafish was checked every weekday, focusing on their swimming movement and vertical position, movements of the mouth and opercular flap associated with gill respiration, feed aggressiveness, and body surface condition.

### Egg collection and exposure of embryos to lead

Adult zebrafish of a mating group were transferred from a rearing tank to a spawning tank (polystyrene cuboid tank with a capacity of 1 L) in the circulating water system in the evening prior to egg collection. Eggs from natural breeding, which occurs at lighting time in the morning, were collected immediately after spawning and cultured at 28.5°C in a 100 mm cell culture dish containing 30 ml of egg culture water (0.006% NaCl; 0.00025% methylene blue in deionized water) in a humidified incubator.

At 6 h post fertilization (hpf), unfertilized eggs and abnormally developed embryos were removed, and healthy embryos were evenly divided into exposure and control groups at random. The embryos were transferred to 6-well plates filled with 10 ml of egg culture water alone or containing 100 ppb (10 μg/dl) of Pb (lead acetate: Pb(CH_3_COO)_2_, Yoneyama Yakuhin Kogyo, Osaka, Japan, CAS No: 6080-56-4, purity: ≥ 99.0%). The number of embryos was adjusted not to exceed 30 per well. Egg culture water with or without Pb was replaced once a day (48 and 72 hpf) until embryos/larvae were used in subsequent experiments.

After the exposure period (6–72 hpf), a portion of the control and the Pb-exposed larvae was rinsed twice with the egg culture water and transferred to the polystyrene cuboid 1 L tank, to avoid hypoxia, and maintained under a 14/10-h light/dark cycle at 28.5°C until 7 dpf. The tank water was replaced every alternate day.

### Determination of viability, hatching rate, and body length

The viability of the embryos/larvae was determined by the presence or absence of heartbeat and blood flow, and hatching was defined as a complete egression or protrusion of a part of the body (mostly the tail) from the chorion. The embryos/larvae were observed under a stereomicroscope SZX9 (Olympus, Tokyo, Japan) with a hot stage that could keep the observation objects at 28.5°C (Tokai Hit, Shizuoka, Japan), and viability and hatching rate were determined at 24, 48, and 72 hpf. The determination of viability and hatching rate was repeated six times at each time point using independently collected embryos from different mating groups (20–40 control and Pb-exposed embryos/larvae in each trial).

To avoid the time lag and obtain accurate body length data at each time point (24, 48, and 72 hpf), the embryos/larvae were briefly fixed with cold 4% paraformaldehyde in phosphate buffered saline (PBS) at 4°C for 1 h with continuous rotation and then stored in PBS at 4°C until the measurement. Fixed embryos/larvae were photographed using a stereomicroscope Stemi305 (Zeiss, Oberkochen, Germany), and body length was measured using Labscope software (Zeiss, RRID:SCR_021768). Body length measurements were repeated three times for each time point using independently collected embryos from different mating groups (10 control and Pb-exposed embryos/larvae in each trial).

### Photography of live embryos/larvae

To capture the images, live embryos/larvae were anesthetized with 0.01% tricaine methanesulfonate (MS-222) in egg culture water up to a minute, depending on the efficacy of anesthesia, and then mounted on a cold 4% methylcellulose solution on a glass slide. Photography was performed using a stereomicroscope Stemi305 (Zeiss) at 24, 48, and 72 hpf and 7 dpf. Live photography was repeated three times for each time point using independently collected embryos from different mating groups (5 control and Pb-exposed embryos/larvae in each trial).

### Measurement of reactive oxygen species levels

The levels of ROS in control and Pb-exposed embryos/larvae were measured using 2′,7′-dichlorodihydrofluorescein diacetate (DCFH-DA) method. At 24, 48, and 72 hpf, the embryos whose chorion was removed and/or larvae were placed into 6-well plates filled with 10 ml of the egg culture water containing DCFH-DA at a final concentration of 20 μg/ml and incubated at 28.5°C for 30 min in dark. The embryos were then rinsed twice with egg culture water and lysed in RIPA buffer (50 mM Tris-HCl pH 7.4, 150 mM NaCl, 1% Triton X-100, 0.5% sodium deoxycholate, 0.1% sodium dodecyl sulfate, and 1 mM ethylenediamine-*N′,N′,N′,N′*-tetraacetic acid). The fluorescence of the lysates was measured (excitation at 480 nm, emission at 530 nm) using a microplate reader SH-9000Lab (CORONA ELECTRIC, Ibaraki, Japan). The ROS measurement was repeated three times for each time point using independently collected embryos from different mating groups, and three pools of embryos/larvae from the same mating group, which consisted of 13–23 embryos/larvae, were used in each trial. The results of each measurement are represented as the mean of the data from these three pools.

### RNA extraction and real-time quantitative PCR

Total RNA was extracted from the control and Pb-exposed embryos/larva using the RNeasy Plus Mini Kit (QIAGEN, Hilden, Germany), and genomic DNA was additionally removed using an RNase-free DNase set (QIAGEN). RNA extraction was performed four times for 24 and 48 hpf and seven times for 72 hpf embryos/larvae using independently collected embryo pools from different mating groups (30–50 control and Pb-exposed embryos/larvae in each trial). RNA was reverse-transcribed into complementary DNA (cDNA) using ReverTra Ace qPCR RT Master Mix (TOYOBO, Osaka, Japan). Reverse transcription was performed in duplicate for each RNA sample. The cDNA samples were subjected to quantitative real-time PCR (qRT-PCR) using Power SYBR Green PCR Master Mix in the StepOne real-time PCR system (Thermo Fisher Scientific, Waltham, MA, United States). The primers used are listed in Supplementary Table S1. To validate the stability of expression of housekeeping genes and select appropriate internal controls for each time point, the threshold cycle (CT) values of *actin, beta1* (*actb1*), *beta-2-microglobulin* (*b2m*), and *glyceraldehyde-3-phosphate dehydrogenase* (*gapdh*) were obtained by qRT-PCR eight times for each time point with the use of two sets of independently reverse transcribed cDNAs from each of the four different RNA samples (Supplementary Table S2). The results of each qRT-PCR analysis are presented as the mean of the data from the trials in duplicate. The duplicate qRT-PCR sets were repeated four or seven times for each time point using the cDNAs described above.

### Statistical analysis

Values are expressed as the mean ± standard deviation (SD). Statistical significance was determined by a Student’s *t*-test or one-way analysis of variance (ANOVA) followed by Tukey-Kramer honest significant difference test using JMP Pro 13 (SAS Institute, Cary, NC, United States, RRID:SCR_014242). A value of *p* < 0.05 was considered to be statistically significant. There were no data excluded from statistical analysis.

## Results

### Lead shortens hatching period and body length

**FIGURE 1:**
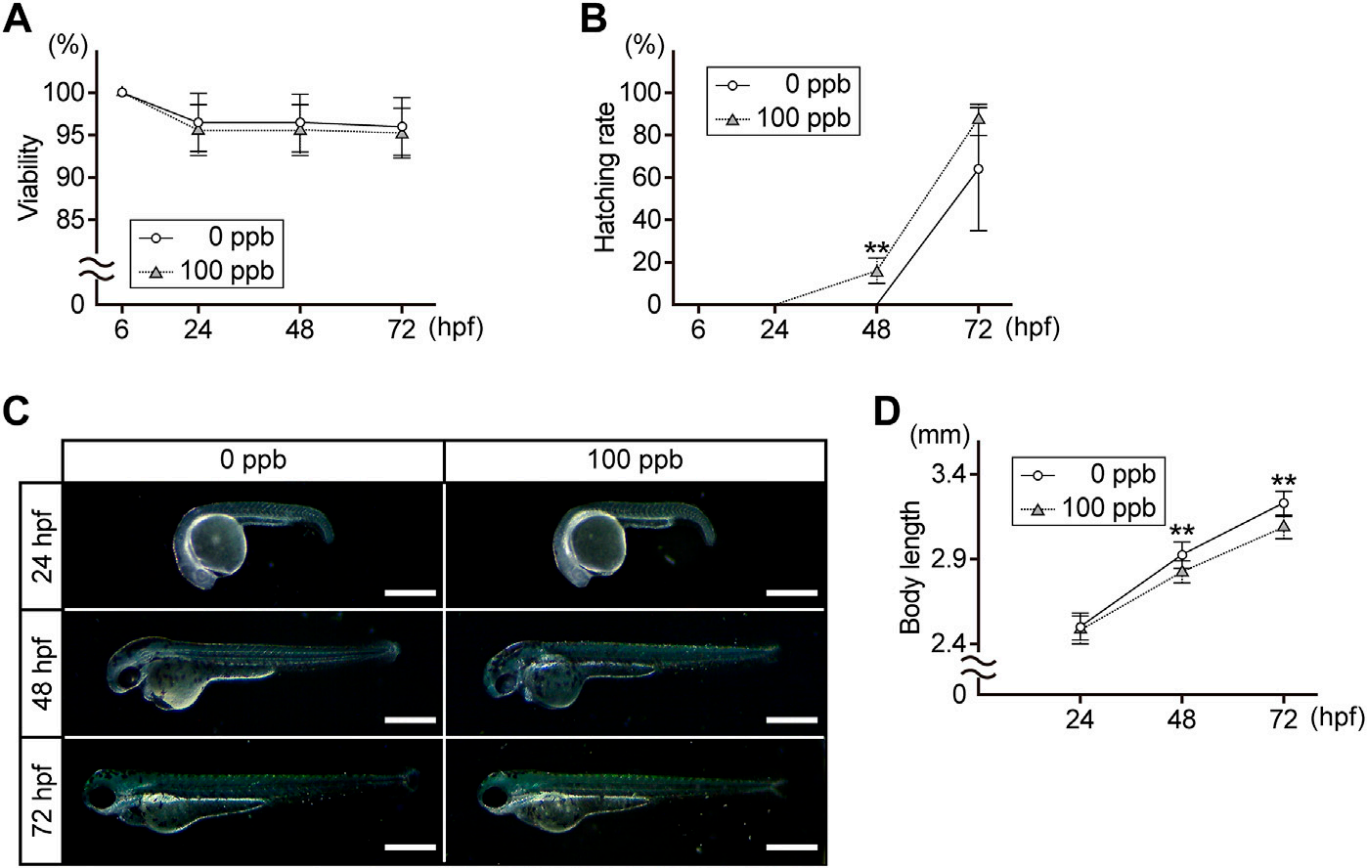
Viability, hatching rate, external morphology, and body length of the Pb-exposed (100 ppb Pb) and the control (0 ppb Pb) zebrafish embryos/larvae. **(A)** viability, **(B)** hatching rate, and **(D)** body length are shown in the line charts. Circles (control) and triangles (Pb-exposed) with error bar represent the mean ±SD. **(C)** photographs are representative of three experiments and oriented anterior to the left and dorsal to the top. Scale bar = 500 μm. **(A,B,D)** statistical significance between control and Pb-exposed groups at each time point was calculated by the Student’s t-test. **p < 0.01. **(A,B)** number of trials = 6, *n* = 20–40. **(D)** number of trials = 3, *n* = 10.

First, we compared the viability, hatching rate, external morphology, and body length of the embryos/larvae exposed to 100 ppb Pb with the controls at 24, 48, and 72 hpf. There was no difference in viability between the control and Pb-exposed groups at any time point (Figure 1A). Approximately 16% of the Pb-exposed embryos hatched at 48 hpf, whereas control embryos did not. At 72 hpf, the hatching rate of the Pb-exposed groups was higher than that of the control groups (87.5 ± 7.4% vs. 65.0 ± 29.3%) (Figure 1B). Superficially evident morphological alterations were not observed in the Pb-exposed embryos/larvae compared to the controls at any time point (Figure 1C); however, the body length of the Pb-exposed embryos/larvae was slightly (3%–4%) shorter than that of the controls at 48 and 72 hpf (Figure 1D).

### Lead induces abnormalities later in development

**FIGURE 2:**
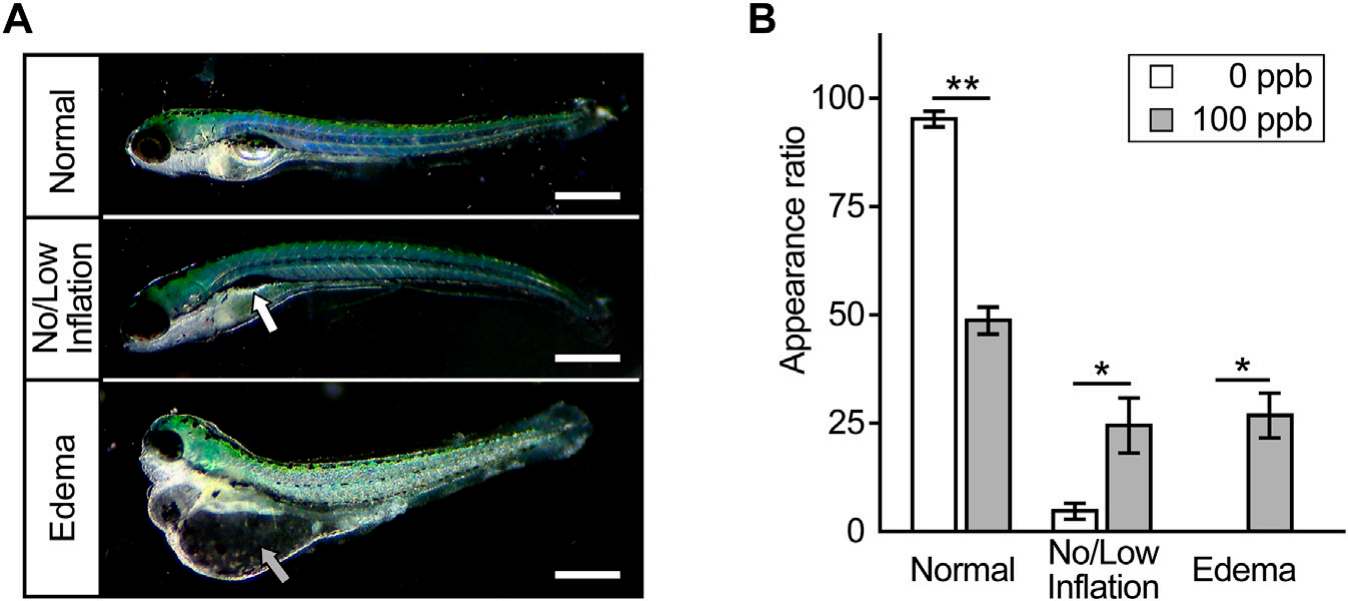
Anomalies appeared later in development. **(A)** Typical microscopic images of zebrafish larvae at 7 dpf that were exposed to Pb during 6–72 hpf. Photographs are representative of three experiments and oriented anterior to the left and dorsal to the top. Scale bar = 500 μm. White and gray arrow indicates incompletely inflated swim bladder and edema, respectively. **(B)** The appearance rate (%) of inflation defects of swim bladder and edema is shown in the bar graph representing the mean ± SD. Statistical significance between control and Pb-exposed groups at each time point was calculated by the Student’s t-test. *p < 0.05, **p < 0.01.

Next, we investigated whether exposure of embryos to Pb during 6–72 hpf, included within mammalian developmental stages from implantation to birth, causes developmental defects at later stages of development. At 7 dpf, the larvae exposed to Pb until 72 hpf presented no or low inflation of the swim bladder (Figure 2A). These inflation defects appeared in approximately 25% of Pb-exposed larvae, in contrast to 5% of control larvae (Figure 2B). In addition, over 25% of the Pb-exposed larvae showed prominent edema in the pericardium, yolk sac, and head, all of which were not observed in the control larvae (Figures 2A,B). Of note, due to the deformity of the abdomen accompanying edema formation, the number of inflation defects of the swim bladder simultaneously occurred in edematous individuals was unclear. Thus, we could not determine the overlap between these defects when calculating the appearance rate.

### Lead alters reactive oxygen species level

**FIGURE 3:**
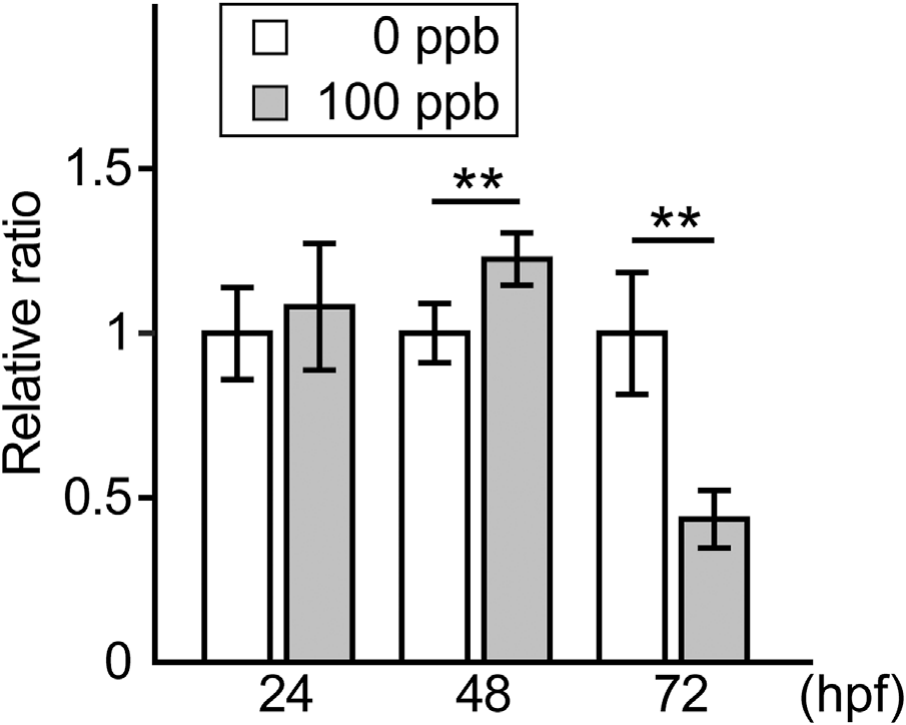
ROS level in the Pb-exposed (100 ppb Pb) and the control (0 ppb Pb) zebrafish embryos/larvae. Relative ratio of ROS amount per embryo/larva to the mean of control at 24, 48, and 72 hpf are shown in the bar graph representing the mean ± SD. Statistical significance was calculated by the Student’s *t*-test compared between control and Pb-exposed groups at each time point. **p < 0.01. Number of trials = 3, *n* = 13–23. Each trial consisted of three n.

We then examined ROS levels in Pb-exposed embryos/larvae and compared them to those in the controls. The relative ratio of ROS per embryo/larva in the Pb-exposed groups to the controls was similar at 24 hpf, slightly (approximately 1.2 times) but significantly higher at 48 hpf, and then reduced to less than half at 72 hpf (Figure 3).

### Lead induces oxidative stress response

**FIGURE 4:**
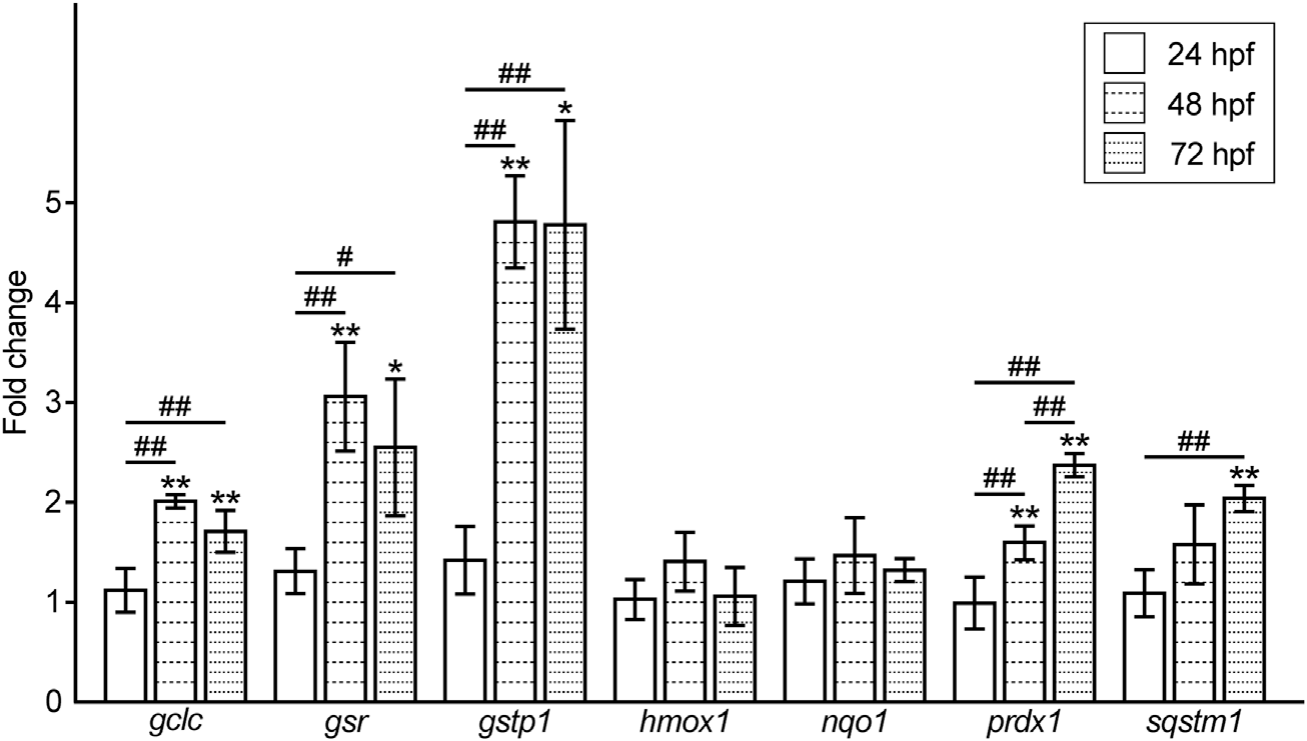
Fold changes in the expression level of oxidative stress response-related genes in the Pb-exposed (100 ppb Pb) zebrafish embryos/larvae compared to the control (0 ppb Pb). The values of the fold changes at 24, 48, and 72 hpf are shown in the bar graph representing the mean ± SD. Statistical significance between control and Pb-exposed groups at each time point was calculated by the Student’s t-test. *p < 0.05, **p < 0.01. Statistical significance among time point was calculated by one-way analysis of variance (ANOVA) followed by Tukey-Kramer honest significant difference test. #*p* < 0.05, ##*p* < 0.01. Number of data = 4 (mean of the data from duplicated experiments using independently synthesized cDNAs with four different RNA samples), *n* = 30–50.

Accordingly, we examined the expression levels of oxidative stress response-related genes in Pb-exposed embryos/larvae and compared them to the controls. The expression levels of antioxidant enzymes, *heme oxygenase 1* (*hmox1*) and *NAD(P)H dehydrogenase quinone 1* (*nqo1*) in the Pb-exposed groups were almost the same as those in the controls at all the time points tested. In contrast, the genes involved in the antioxidant role of glutathione, *glutamate-cysteine ligase, catalytic subunit* (*gclc*), *glutathione reductase* (*gsr*), and *glutathione s transferase p1* (*gstp1*), were upregulated at 48 hpf and maintained at high levels at 72 hpf, whereas *gclc* and *gsr* showed a slight reduction at this time point. Another antioxidant enzyme, *peroxiredoxin 1* (*prdx1*), and an oxidative stress regulator, *sequestosome 1* (*sqstm1*), were upregulated in a time-dependent manner (Figure 4).

### Lead induces endoplasmic reticulum stress response

**FIGURE 5:**
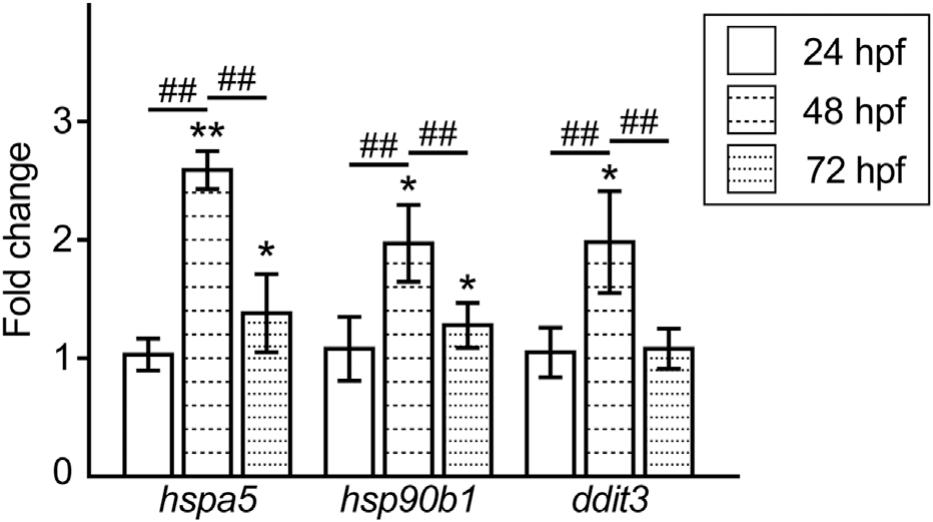
Fold changes in the expression level of the ER stress response-related genes in the Pb-exposed (100 ppb Pb) zebrafish embryos/larvae compared to the control (0 ppb Pb). The values of the fold changes at 24, 48, and 72 hpf are shown in the bar graph representing the mean ± SD. Statistical significance between control and Pb-exposed groups at each time point was calculated by the Student’s t-test. *p < 0.05, **p < 0.01. Statistical significance among time point was calculated by one-way ANOVA followed by Tukey-Kramer honest significant difference test. ##*p* < 0.01. Number of data ≥ 4, *n* = 30–50.

Moreover, we examined the expression levels of ER stress response-related genes in the Pb-exposed embryos/larvae and compared them to those of the controls. The expression of the ER-resident chaperones, *heat shock protein 5* (*hspa5*) and *heat shock protein 90 beta (grp94) member 1* (*hsp90b1*), in the Pb-exposed groups was upregulated at 48 hpf and decreased but was slightly higher than that in the controls at 72 hpf. In contrast, the ER stress-dependent apoptotic factor *DNA-damage-inducible transcript 3* (*ddit3*) was upregulated at 48 hpf and then completely reduced to the control level at 72 hpf (Figure 5).

## Discussion

### Developmental effects

In the present study, we aimed to provide direct experimental evidence of adverse effects of Pb exposure below the occupational regulatory standard concentrations on embryonic/larval development using a zebrafish model. We exposed zebrafish embryos to 100 ppb Pb, which meets the representative current occupational regulations for BLL, during the developmental period included within mammalian implantation to birth. The exposed embryos/larvae hatched earlier and were significantly shorter in body length than the controls at 48 and 72 hpf without superficially evident morphological alterations or death. These data indicate that embryos exposed to 100 ppb Pb tend to hatch precociously and suggest that exposure to low concentrations of Pb causes premature birth and/or growth retardation as indicated by the short body length, which may be associated with low birth weight. These findings are in agreement with previous epidemiological information that a very low level of BLL (below 5 μg/dl) can cause adverse effects on fetal development including growth retardation and low birth weight (NTP, 2012). In addition, a recent study using zebrafish revealed that exposure of embryos/larvae to a series of low concentrations of Pb, including almost the same concentration as we used, increases hatching rate at 72 hpf and shortens body length at 4 dpf (Wang et al., 2022). They also showed that exposure of embryos/larvae to 0.5 μM Pb (104 ppb Pb, approximately equal to our test concentration) does not affect survival rate (Wang et al., 2022). Although there are some small differences in the results and experimental conditions from those of our study, these data strongly support our findings. On the other hand, contrary to our data, in which evident morphological alterations were not observed during the exposure period, the malformation rate increased at 2 dpf and remained at the same level until 6 dpf in the previous study (Wang et al., 2022). In addition, Park et al. (2020) showed that exposure to very low concentration of Pb (∼17 ppb) decreases survival rate and induces malformations at 2 dpf and later. The reason for these discrepancies is unclear; however, a possible explanation is the differences in zebrafish strain, sensitivity of fish to Pb exposure, start time of exposure, and/or in judgement criteria for malformation.

Additionally, we showed that larvae exposed to Pb until 72 hpf and then cultured until 7 dpf without Pb showed developmental anomalies, including no or low inflation of the swim bladder and edema formation in the head, pericardium, and yolk sac. Previous reports have also shown that exposure of zebrafish embryos/larvae to Pb results in the absence of a swim bladder and/or the formation of pericardial and yolk sac edemas; however, these abnormalities were observed in larvae continuously exposed to more than four times, up to 480 times, higher concentrations than those used in this study (Yin et al., 2017; Jin et al., 2019; Liu et al., 2019; Wang et al., 2022). Inflation problems of the swim bladder and edema formation are non-specific and common malformations that occur regardless of the type of exposed toxicant. Therefore, we could not identify the potential site of Pb action in developmental events only from these abnormalities; however, this finding can be evidence of the adverse effects that occurred as a result of the fetal exposure to trace amounts of Pb. From the late 1980s to the 1990s, Barker et al. established a hypothesis which suggested that intrauterine growth retardation, low birth weight, and premature birth have a causal relationship with the origins of hypertension, coronary heart disease, and non-insulin-dependent diabetes in middle age (Barker and Osmond, 1986; Barker et al., 1989; Barker, 1990). Subsequently, Gluckman and Hanson (2006) extended this hypothesis to the idea that the embryonic and early childhood environment determines future health and disease susceptibility, designated as the Developmental Origins of Health and Disease (DOHaD) hypothesis or DOHaD theory. The DOHaD theory is now widely accepted as a very important concept regarding health problems. The number of previous experimental studies on the effects of Pb from the viewpoint of DOHaD is not large and biased toward transcriptomics and epigenetic alterations including DNA methylation status (Lee et al., 2018; Shiek et al., 2021), which investigate the cause of but not the result of adverse health effects induced by Pb exposure. In this context, our finding of developmental anomalies at 7 dpf is a new example supporting the DOHaD theory.

### Oxidative stress

Exposure of zebrafish embryos/larvae to 100 ppb Pb increased both ROS levels and the expression levels of oxidative stress response-related genes, including *gclc*, *gsr*, *gstp1*, *prdx1*, and *sqstm1* at 48 hpf. In contrast, ROS level at 72 hpf was reduced to less than half of the control level, whereas the expression of these genes was maintained at a high level. Such discordance may be due to the exceeding antioxidation activity triggered by weakly induced oxidative stress, which appeared as the slight elevation of the ROS level at 48 hpf. Because of our temporally broad sampling plan, it is unclear to what extent ROS levels increase in zebrafish embryos/larvae following exposure to 100 ppb Pb; however, a significant decrease in ROS at 72 hpf can be explained by this hyper-reactivity to ROS generation. It is possible that this dynamic change in ROS level, not only the increase but also the subsequent decrease, may be involved in the mechanism underlying the toxicity caused by trace amounts of Pb.

The expression of *gclc*, *gsr*, *gstp1*, *prdx1*, and *sqstm1*, but not *hmox1* and *nqo1*, clearly increased at 48 and 72 hpf. All of these genes act as downstream (*gclc, gsr, gstp1, prdx1, hmox1,* and *nqo1*) (Venugopal and Jaiswal, 1996; Alam et al., 1999; Ikeda et al., 2002; Sekhar et al., 2002; Kim et al., 2007; Harvey et al., 2009; Nakajima et al., 2011; Mills and Gallagher, 2017) and upstream (*sqstm1*) (Komatsu et al., 2010; Bellezza et al., 2018) genes of *nuclear factor-E2-related factor 2* (Nrf2), a key regulatory transcription factor for oxidative stress response-related genes (Bellezza et al., 2018). In these genes, *gclc*, *gsr*, and *gstp1* were characteristically upregulated in this study and have been known to participate in the process of reduced glutathione (GSH)-dependent antioxidation as enzymes (Lu, 2009; Laborde, 2010; Couto et al., 2016). On the other hand, *hmox1* and *nqo1*, which were not affected by Pb exposure in expression, act in distinct and GSH-independent antioxidation mechanisms (Dinkova-Kostova and Talalay, 2010; Loboda et al., 2016; Vanella et al., 2016; Ross and Siegel, 2017). These data suggest the possibility that GSH primarily promotes the antioxidative activity against ROS induced by low-dose Pb exposure in zebrafish. In accord with this idea, many authors have reported significant increases or decreases in GSH levels in various fish species exposed to Pb (Lee et al., 2019). On the other hand, contrary to our data, a recent study reported that exposure of zebrafish embryos to Pb from 2.5 hpf to 98.5 h increases the expression of *hmox1* as well as *gst*, at the same concentration as we tested (Kataba et al., 2022). In addition, they also showed that pulse exposure of 120 hpf zebrafish larvae to quite low concentration (3 μg/L) of Pb induces *hmox1* expression (Kataba et al., 2020). This discrepancy suggests that Hmox1 may also play a role in response to oxidative stress caused by Pb, depending on the exposure conditions, zebrafish strain, and/or sensitivity of fish to Pb exposure.

### Endoplasmic reticulum stress response

We observed transient and evident upregulation of the ER-resident chaperones *hspa5* and *hsp90b1* and the ER stress-dependent apoptotic factor *ddit3* at 48 hpf. The expression of these genes seems to be increased in parallel with the increment of ROS, because ER stress has been interactively linked to intracellular production of ROS; that is, ER stress results in the accumulation of ROS and simultaneously ROS accelerate the dysfunction of ER (Cullinan and Diehl, 2006; Cao and Kaufman, 2014; Chaudhari et al., 2014). In addition, we previously showed that elimination of toxicant-induced ROS by treatment with ROS scavenger ameliorates the ER stress state (Komoike and Matsuoka, 2016). In line with these previous findings, our data suggest that the decrease in ROS lowers the expression of ER stress-related genes, while at the same time, cytoprotective *hspa5* and *hsp90b1*, but not cytopathic *ddit3*, at slightly higher levels than the control, contribute to the elimination of ROS at 72 hpf. Although the relationship between Pb exposure and ER stress and the related responses have been well documented, Qian et al. (2000, 2005) provided interesting evidence that Pb directly binds to the GRP78 (a synonym of Hspa5) protein, increases its protein level, and induces its compartmentalized redistribution, indicating that Pb can directly induce ER stress. It is therefore possible that Pb also directly binds to the Hspa5 protein in zebrafish embryos/larvae; however, if so, Pb may maintain the expression of *hspa5* at higher levels during the exposure period. Our finding that *hspa5* in the Pb-exposed larvae was reduced at 72 hpf compared to 48 hpf is contradictory to this idea, and thus suggests that ER stress in the Pb-exposed zebrafish embryos/larvae is mainly induced via a non-direct and ROS-dependent mechanism.

### Lead concentration

Previous studies using zebrafish have indicated that Pb in exposure water accumulates in the zebrafish body with a wide range of bioconcentration ratios, approximately 1.5–900 times (Zhang et al., 2011; Tu et al., 2017; Liu et al., 2019; Zhao et al., 2020). Of the various metals, Pb is one of the most accumulative toxic metals due to its property of easily binding to oxygen and sulfur atoms in proteins to form a stable complex (Lee et al., 2019). The bioconcentration ratio may vary depending on the exposure conditions, including the concentration, duration, developmental timing, and frequency of water replacement. It is therefore impossible to compare the data obtained under our experimental conditions with these previous results; however, it is natural to think that Pb also accumulated in the bodies of exposed embryos/larvae in this study. Although we selected 100 ppb (10 μg/dl) as the exposure concentration with reference to the representative current occupational regulations for BLL, the actual BLL in Pb-exposed zebrafish embryos/larvae is thought to be higher than originally intended. However, because zebrafish embryos/larvae are too small to divide into organs, the content of Pb has been compelled to be expressed as Pb (ng)/wet weight of the whole body (g) in most previous studies. It is unclear how far the average Pb weight per unit weight of whole-body tissue mixture correlates with BLL, and thus, does not seem to be a suitable alternative to BLL. In consideration of these circumstances, investigation of the developmental effects of Pb on zebrafish at concentrations lower than 100 ppb will be needed in future studies.

## Conclusion

Our findings using a zebrafish model indicate that exposure of embryos to trace amounts of Pb below the occupational regulatory standard concentrations induces a transient increase in oxidative and ER stresses and results in weak hypotrophy around the hatching period and subsequent morphological abnormalities in larval development. However, the causal relationships between oxidative and ER stresses, weak hypertrophy, and malformations remain unclear. Further experimental demonstrations of the mechanisms responsible for Pb toxicity are required. Finally, this study may provide clues for further exploration of the total health effects of Pb exposure, and the zebrafish is a good animal model for the investigation of developmental toxicity of pollutants.

## Data Availability

The raw data supporting the conclusions of this article will be made available by the authors, without undue reservation.
