# Supplementary material for: Developmental adverse effects of trace amounts of lead: Evaluation using zebrafish model
Source: Front Pharmacol. 2022 Sep 21;13:1014912. doi: 10.3389/fphar.2022.1014912 (PMC9532946; doi:10.3389/fphar.2022.1014912)
Supplement: Supplementary file 1 [file DataSheet1.docx]

Supplementary Material

**Supplemental Table S1**: genes analyzed by real time quantitative PCR.

| Gene | Gene ID | Forward Primer | Reverse Primer |
| --- | --- | --- | --- |
| *gclc* | [326857](https://www.ncbi.nlm.nih.gov/gene/?term=326857) | GCTTACGTGGTGTTTATCGTTCTG | GACAACGGAATGAGGAAATCCA |
| *gsr* | [553575](https://www.ncbi.nlm.nih.gov/gene/?term=553575) | CGAGGAGAGCGGCTGAAC | ACCTCCGAGTCTGTGACTTTCG |
| *gstp1* | [79381](https://www.ncbi.nlm.nih.gov/gene/?term=79381) | AGCAACTTTGAAACGCACTTCA | TGACTGCGAAGTATGTGAGTGTGT |
| *hmox1a* | [791518](https://www.ncbi.nlm.nih.gov/gene/?term=791518) | CATCCCAAAGTCTGTCAAAAACC | CAACGTGATGCCCACTCCTA |
| *nqo1* | [322506](https://www.ncbi.nlm.nih.gov/gene/?term=322506) | AGAGGCTGGGTGGTGTGTTT | CCTGCTTGAAAGCTAAGGTCAAA |
| *prdx1* | [541344](https://www.ncbi.nlm.nih.gov/gene/?term=541344) | AGACTTGAGCACGACCTATTTAAACA | TCCGAGAGTGAATGAATGAAAGTG |
| *sqstm1* | [406452](https://www.ncbi.nlm.nih.gov/gene/?term=406452) | AGAGCAAAGGCCTGCACAA | CGAGGGAGCCACTCAAACAC |
| *hspa5* | [378848](https://www.ncbi.nlm.nih.gov/gene/?term=378848) | ACAGCCGCAATGAATTGGA | CTCTTTATCCCCGATCTGGTTCT |
| *hsp90b1* | [386590](https://www.ncbi.nlm.nih.gov/gene/?term=386590) | CCCAGCACATGTGGGAATCT | TCCACGTGGGTCCTCGAT |
| *ddit3* | [561924](https://www.ncbi.nlm.nih.gov/gene/?term=561924) | CCGCCCGTTCACCAATC | AGGTGTTCTCCGTGGTTCGT |
| *actb1* | [57934](https://www.ncbi.nlm.nih.gov/gene/?term=57934) | TGCCCCTCGTGCTGTTTT | TCCCATGCCAACCATCACT |
| *b2m* | [30400](https://www.ncbi.nlm.nih.gov/gene/?term=30400) | CTGCTGAAGAACGGACAGGTT | CTGCCAGCCCTTTTCGAA |
| *gapdh* | [317743](https://www.ncbi.nlm.nih.gov/gene/?term=317743) | CGGATTCGGTCGCATTG | GGCCACGATCTCCACTTTCTT |

Note: All primers were used with reference to our previous study (Komoike and Matsuoka, 2019).

**Supplemental Table S2**: expression stability of housekeeping genes.

| hpf | Gene Name | Ct^Con^  Mean ± SD | Ct^Pb^  Mean ± SD | \| Mean  (Ct^Pb^ − Ct^Con^) \| | SD  (Ct^Pb^ − Ct^Con^) |
| --- | --- | --- | --- | --- | --- |
| 24 hpf | *actb1* | 21.792 ± 0.155 | 21.669 ± 0.388 | 0.124 | 0.468 |
|  | *b2m* | 33.141 ± 0.570 | 32.734 ± 0.362 | 0.407 | 0.369 |
|  | ***gapdh*** | 25.690 ± 0.421 | 25.572 ± 0.562 | 0.118 | 0.224 |
| 48 hpf | ***actb1*** | 20.927 ± 0.646 | 20.708 ± 0.720 | 0.219 | 0.353 |
|  | *b2m* | 32.991 ± 0.464 | 31.296 ± 0.725 | 1.695 | 0.846 |
|  | *gapdh* | 24.478 ± 0.441 | 24.406 ± 0.578 | 0.072 | 0.602 |
| 72 hpf | ***actb1*** | 19.895 ± 0.542 | 19.885 ± 0.586 | 0.010 | 0.302 |
|  | *b2m* | 29.965 ± 0.826 | 29.081 ± 0.682 | 0.884 | 0.352 |
|  | *gapdh* | 21.056 ± 0.742 | 20.505 ± 0.540 | 0.552 | 0.346 |

Notes: number of data = 8 (obtained from two cDNA sets synthesized by duplicated reverse transcription with four independently collected RNA samples for each time point). Ct: threshold cycle; SD: standard deviation; Con: control groups (0 ppb Pb); Pb: exposed groups (100 ppb Pb). Genes in bold were used as an internal control at each time point.

**Reference**

Komoike, Y., and Matsuoka, M. (2019). In vitro and in vivo studies of oxidative stress responses against acrylamide toxicity in zebrafish. *J Hazard Mater* 365**,** 430-439. doi: <https://doi.org/10.1016/j.jhazmat.2018.11.023>.
